# Mesenchymal Stromal Cell Rejuvenation Strategies to Enhance Clinical Translation in Cell Therapy

**DOI:** 10.1111/acel.70672

**Published:** 2026-08-17

**Authors:** Astrid Sodomaco, Irene Bernardi, Aretha Rambaldi, Pierluigi Lombardi, Maura Rossi, Francesca Paris, Lucia Manzoli, Stefano Ratti, Francesco Alviano

**Affiliations:** ^1^ Cellular Signalling Laboratory, Anatomy Center, Department of Biomedical Sciences (DIBINEM) University of Bologna Bologna Italy; ^2^ Department of Medical and Surgical Sciences (DIMEC) University of Bologna Bologna Italy

**Keywords:** cell therapy, cellular senescence, epigenetic reprogramming, mesenchymal stromal cell, oxidative stress, perinatal stem cell, stem cell aging

## Abstract

Cell therapies represent a promising frontier in modern medicine. Mesenchymal stromal cells (MSCs) constitute a valuable source due to their minimal ethical concerns, low immunogenicity, negligible tumorigenicity in vivo, and robust paracrine activity mediated by the secretion of anti‐inflammatory and angiogenic factors. Despite these advantages, MSCs undergo rapid replicative senescence accompanied by a progressive loss of stemness markers and functional potency. In this context, cellular rejuvenation has been emerging as a pivotal strategy to restore their original biological features. These approaches aim to modulate the hallmarks of aging; as these processes are highly interconnected, multiple and complementary targets have been proposed. This review aims to discuss and analyze the main rejuvenation strategies reported in the literature for MSCs, with particular attention to approaches that can enhance their functionality and translational potential. In this context, the review focuses on five key categories of interventions: epigenetic modifications, metabolic reprogramming, modulation of cellular senescence, telomere biology, and extracellular vesicles. These strategies represent the fundamental axes through which cellular aging can be counteracted and the efficacy of cell‐based therapies improved.

## Graphical abstract

MSC rejuvenation strategies and functional outcomes. Aging‐associated alterations in MSCs, including increased IL‐6/IL‐8 secretion, ROS accumulation, epigenetic drift, and mitochondrial dysfunction, impair stemness. Rejuvenation approaches, epigenetic modulation (VPA, AZAc, OSKM, TERT), metabolic interventions (resveratrol, calcitriol, NMD, asprosin), and senolytic therapies (dasatinib, quercetin, fisetin) restore self‐renewal, improve redox balance, and extend culture lifespan, enhancing therapeutic potential in.

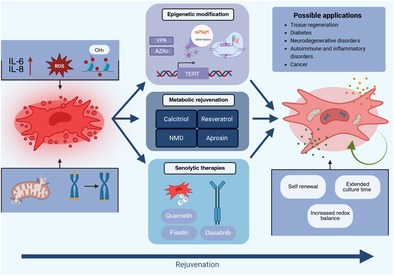

## 1 Introduction

### 1.1 Cell Therapies and Mesenchymal Stromal Cells (MSCs)

Cell therapies have emerged as a new major frontier and represent a cornerstone shift in biotechnology, establishing the “third pillar” of therapeutic intervention in modern medicine. Unlike conventional small molecules, cells can perform complex biological functions and respond to biological cues dynamically, making them an advantageous resource (Zhao et al. 2020; Fischbach et al. 2013).

Among various stem cell types, MSCs are particularly attractive, as they can be sourced from routine surgical procedures, exhibit unique biological properties, and raise few to no ethical concerns (Zhang and Lai 2020).

MSCs are multipotent cells found in several tissues, including bone marrow, adipose tissue, muscle, peripheral blood, and perinatal tissues and are capable of differentiating into various lineages such as bone, cartilage, and adipose tissue. MSCs are characterized by the expression of CD73, CD90, and CD105, and the absence of hematopoietic markers. They have shown good prospects and reshaped the clinical landscape of tissue repair and regeneration, as well as anti‐tumor strategies (Mei et al. 2024; Brown et al. 2019).

A key advantage of MSCs is their homing ability to damaged tissues, which can be either systematic or unsystematic. Unsystematic homing refers to direct local transplantation to a specific damaged site while systematic homing refers to the active migration of MSC from peripheral circulation to the target site guided by homing promoting factors released by the damaged tissue. Homing is particularly interesting for clinical applications since MSCs continuously repair damaged tissue through brief interactions and paracrine signaling rather than long‐term engraftment relying on a “hit and run” mechanism (Mei et al. 2024).

MSCs can also be isolated from perinatal tissues, which are typically discarded as medical waste after birth. In particular, the umbilical cord, amniotic fluid, and fetal membranes represent rich and readily accessible sources of stem cells (Paris et al. 2022). Their recovery from these tissues offers a practical and ethically straightforward source of MSCs with considerable promise for future therapeutic applications.

Perinatal stem cells are particularly attractive due to their immunomodulatory features and their intrinsic immunoprivileged profile. In fact, their very low expression of HLA Class II molecules allows their use in allogeneic transplantation even without full donor‐recipient matching, facilitating clinical application and supporting the development of “off‐the‐shelf” therapeutic products (Le Blanc et al. 2003; Miki 2018). In addition, MSCs, including those isolated from perinatal tissues, exhibit a markedly lower risk of teratoma formation compared with pluripotent stem cells, further reinforcing their safety and suitability for clinical translation (Mei et al. 2024; Naeem et al. 2022).

Despite these advantages, several challenges still limit the clinical translation of MSC‐based therapies. Among them, cellular senescence represents a major concern; however, culture‐induced replicative senescence and in vivo aging‐associated senescence should be considered distinct, although partially overlapping, processes. The former mainly results from repeated population doublings and manufacturing‐related stress during ex vivo expansion, whereas the latter reflects donor age, tissue‐specific niche alterations, chronic inflammation, and metabolic or oxidative stress occurring in vivo. This distinction is clinically relevant because both donor‐related aging and culture‐induced senescence may independently affect MSC potency, batch consistency, differentiation potential, immunomodulatory activity, and therapeutic efficacy.

**FIGURE 1:**
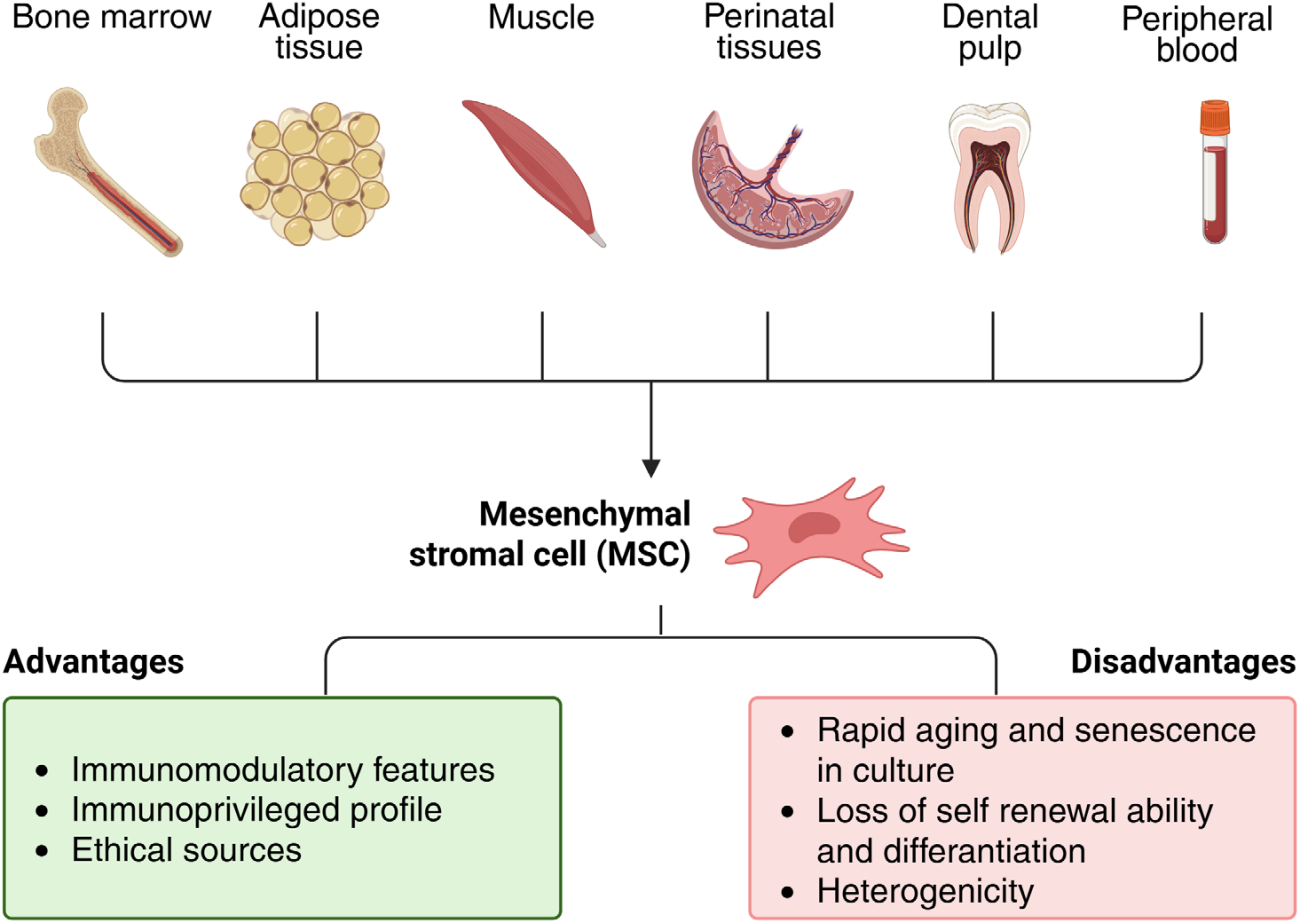
Tissue sources, biological properties, and functional limitations of mesenchymal stromal cells (MSCs). MSCs can be isolated from multiple adult and perinatal tissues, including bone marrow, adipose tissue, skeletal muscle, umbilical cord, placenta, amniotic fluid, dental pulp, and peripheral blood. Across sources, MSCs display conserved immunomodulatory functions and a relative immune‐privileged phenotype, supporting their therapeutic use and ethical accessibility. However, prolonged in vitro expansion induces replicative senescence, leading to reduced self‐renewal capacity and impaired differentiation potential.

In addition, MSCs are characterized by substantial heterogeneity related to donor variability, tissue source and manufacturing conditions, which may influence their functional properties, susceptibility to senescence and responsiveness to rejuvenation strategies (Figure 1) (Mei et al. 2024; Brown et al. 2019). These aspects and their translational implications are discussed in greater detail in the following section. As MSCs are playing an increasingly important role in regenerative medicine, overcoming these limitations is a fundamental goal for expanding their clinical use. To counteract the progressive functional decline of cultured cells and preserve epigenetic regulation and transcriptional activity, different approaches are being investigated to rejuvenate cellular function without compromising lineage identity (Wang et al. 2022). In this context, the term “rejuvenation” is interpreted broadly, encompassing interventions that reverse established senescence‐associated features, delay senescence onset during in vitro expansion, or restore key MSC functions even in the absence of complete senescence reversal. Thus, MSC rejuvenation does not necessarily imply a full return to a developmentally young state, but rather a measurable improvement in therapeutically relevant properties, including proliferation, clonogenicity, differentiation potential, immunomodulatory activity, mitochondrial function and secretory profile.

This review is designed to critically examine and synthesize the principal rejuvenation strategies for MSCs reported in the literature (Table 1), with a specific emphasis on approaches capable of improving their functional performance and clinical translatability. Within this framework, particular attention is given to five main intervention domains: epigenetic remodeling, metabolic reprogramming, the regulation of cellular senescence, telomere biology, and extracellular vesicles. Collectively, these strategies constitute the primary mechanistic axes through which cellular aging may be mitigated, ultimately enhancing the therapeutic efficacy of MSC‐based applications.

**TABLE 1:** Overview of current rejuvenation strategies for mesenchymal stromal cells (MSCs), including partial reprogramming, epigenetic modulation, gene editing, telomerase activation, metabolic interventions, senolytic therapies, and extracellular vesicle‐based approaches.

Rejuvenation strategy	Agent or method	Cell type tested	Molecular targets/pathways	In vitro/in vivo	Clinical/preclinical stage	Experimental conditions	Citations
Partial reprogramming	Viral vectors (non‐integrative Sendai virus vectors), nanoparticles, liposomes	Endometrial derived MSCs	OSKM factors	In vitro and in vivo (mice)	Preclinical	2.4 × 10^9^ pvp per 24 well plates (in vitro) – 1.2 × 10^12^ pvp/mL (in vivo)	Cipriano et al. (2024); Saliev and Singh (2024); Lehmann, Canatelli‐Mallat, Chiavellini, et al. (2019)
Epigenetic modifications/small molecules	5‐aza‐2′‐deoxycytidine (AzadC) or 5‐azacitidine (AzaC)	BM‐MSCs, WJ‐MSCs and AD‐MSCs	DNA methylation, HLA‐G1 and HLA‐G3 expression	In vitro	Preclinical	5–20 μM	Teklemariam et al. (2014); Ong et al. (2021); Kornicka et al. (2017)
	Valproic acid (VPA)	UC‐MSCs, CB‐MSCs and WJ‐MSCs	ERK and AKT pathways, H3K18 lactylation	In vitro	Preclinical	10 mM	Teklemariam et al. (2014)
Gene editing	CRISPR‐Cas9	MSCs/Mesenchymal progenitors	p16INK4a, p53, SOX5, HGMB2, H2AZ1, FOXO3	In vivo (mice and macaques)	Preclinical	Viral (AAV, lentivirus) and non‐viral (LNPs, exosomes) delivery; editing efficiency up to > 90%	Jia et al. (2024); Jing et al. (2023); Lei et al. (2025)
Telomere‐based	Viral approaches	PB‐MSCs, UC‐MSCs, AD‐MSCs, PDLSCs	Telomerase activity, TERT promoter	In vitro and in vivo (mice)	Preclinical	In vitro: hTERT and VEGF transfection In vitro: hTERT with SV40 or HPV E6/E7 In vivo: Adeno‐Associated Virus (AAV)	Davidsohn et al. (2019) Amano et al. (2019); Gabandé‐Rodríguez et al. (2020) Cai et al. (2023)
	Non viral approaches (pistachio green pericap extract, L‐carnitine, curcumin, vitamin C, vitamin E, resveratrol, spermidine)	BM‐MSCs (rats) hASCs rASCs DNA sample Human Leukocytes	Telomerase activity hTERT expression TERT activity and SA‐β‐gal activity TERT activity Prevents DNA damage and telomere shortening protecting polyunsaturated fatty acids (PUFAs)	In vitro In vitro In vitro In vivo (human) In vivo (human)	Preclinical Preclinical Preclinical Human observational and cross‐sectional studies Human observational and cross‐sectional studies	Pistachio green pericap extract: 1000, 2000, and 3000 μg/mL L‐carinitine: 0.1, 0.2, and 0.4 mM Curcumin: 1 and 5 μM Vitamin C: ≤ 41.5 mg/day, 41.5–107.75 mg/day, and ≥ 107.75 mg/day Vitamin E: dietary intake levels adequate/inadequate Resveratrol and Spermidine	Askari et al. (2023) Farahzadi et al. (2018) Pirmoradi et al. (2018) Cai et al. (2023); Qun et al. (2009)[65] Opstad et al. (2021); Amano et al. (2019) Gabandé‐Rodríguez et al. (2020)
Metabolic rejuvenation	Calcitriol (Vitamin D)	hMSCs, mouse BM‐MSCs	VDR‐Ezh2‐p16 Axis, quiescence and Stemness Markers (FOXO1a, FOXO3a, FOXO4, NANOG, TxNIP, and TP53 (p53)), Cell Cycle and Senescence Inhibitors (p16, p21), SASP Factors p16/Rb, p53/p21, SIRT1, Nanog, Sox2	In vitro and in vivo (mice)	Preclinical	In vitro: 100 nM (hMSC stimulation) In vitro: 10 nM (mouse BM‐MSC) In vivo: 1 μg/kg three times weekly (administered to rescue Vitamin D‐deficient mice). 0.1 μg/kg three times weekly (administered to prevent bone loss in naturally aging mice)	Klotz et al. (2012); Borojević et al. (2022); Yang et al. (2020)
	Resveratrol	ASCs, PDLSCs, BM‐MSCs	SIRT1, AMPK, NF‐κB, mitochondrial dynamics, mitophagy	In vitro and in vivo (mice and rats)	Preclinical	In vitro: 0.01–5 μM In vivo: resvetratrol‐treated cell aggregates were transplanted either ectopically (mice) or in situ (rat)	Wang et al. (2018); Kornicka et al. (2019)
	Aprosin	BM‐MSCs, ASCs, MSCs	PI3K/Akt‐HIF‐1 pathway, H3K18 lactylation	In vitro and in vivo (mice)	Preclinical	In vitro: 10 ng/mL—10 μg/mL In vivo: 2 × 10^5^ cells in 25 μL of PBS were injected into three peri‐infarct sites in the heart	Zhang et al. (2026)
	Nicotinamide mononucleotide (NMN)	MSCs	NAD+ levels, PARPs, sirtuins (SIRT1, SIRT3, SIRT6, SIRT7)	In vitro and in vivo (mice, rats, invertebrates)	Preclinical	In vitro: CRISPR–Cas9 to generate NMNAT1 knockout cells In vivo: supplementation of NMN (unknown quantities)	Ruszkiewicz et al. (2022)
Senolytic therapies	Dasatinib and Quercetin	UC‐MSCs (preeclampsia patients) Mouse BM‐MSCs	Ephrin receptor, d Src kinase	In vitro and in vivo (mice, humans)	Preclinical and Clinical Preclinical	In vitro: 1 μM each In vitro: 0.2 μM dasatinib+20 μM quercetin or vehicle (0.1% DMSO) In vivo: 5 × 10^5^ cells treated as above	He et al. (2025) Kirkland et al. (2017)
	Quercetin	BM‐MSCs	repetitive element (RE) activation, the RIG‐I RNA sensing pathway, and the SIRT1/PINK1/Parkin‐mediated mitophagy pathway	In vitro and in vivo (mice)	Preclinical	0.1, 1, 10 and 100 μM	Sun et al. (2025)
	Fisetin	AD‐MSCs, HUVECs, BM‐MSCs	SIRT1, BCL‐2/BCL‐XL, PI3K/AKT/mTOR, NF‐κB	In vitro and in vivo (mice)	Preclinical and Clinical (Phase I and II clinical trials underway)	In vitro: 25, 50 and 100 μM In vivo (mice): 100 mg/kg	Zhu et al. (2017); Mullen et al. (2023); Nishimura et al. (2025)
Senomorphic therapies	Rapamycin	UC‐MSCs Placenta‐derived MSCs	Akt/mTOR pathway mTOR	In vitro *and* in vivo (mice) In vitro	Preclinical Preclinical	In vitro: 0, 50, 100, 500, and 900 nM (optimal) in DMSO In vivo: 1.5 × 10^6^ cells treated as above 10 nM	Cao et al. (2022) Sheppard et al. (2024)
	IKK inhibitor	Aged mouse muscle‐derived stem/progenitor cells	NF‐kB	In vitro	Preclinical	In vitro: 5 μM for differentiation, 600 nM for senescence on BM‐MSCs, 250 μM for oxidative stress	Proto et al. (2017)
Extracellular vesicles	EVs, Edaravone, NAC, genetic manipulation	AT‐MSCs (infant and elderly)	SOD1/SOD3, MEK/ERK	In vitro and in vivo (mice)	Preclinical	In vitro: infant AT‐MSC‐derived EVs, 20 μM edaravone, 2 mM NAC, SOD1/SOD3 modulation In vivo: 0.5 × 10^6^ cells/mouse	Khanh et al. (2020)
	Young MSC‐derived EVs	BM‐MSCs	PCNA‐mediated DNA replication/repair	In vitro and in vivo (mouse)	Preclinical	PCNA delivery from young MSC‐derived EVs	Lei et al. (2021)
	hAMSC‐derived EVs (miR‐21‐5p enriched)	Senescent pancreatic beta cells	miR‐21‐5p/IL‐6RA/STAT3/MCU axis	In vitro *and* in vivo (mouse)	Preclinical	In vitro: incubation of senescent β‐cells with hAMSC‐sEVs In vivo: tail‐vein injection of hAMSC‐sEVs	Xiao et al. (2026)
	Laromestrocel (allogeneic BM‐MSCs)	Human patients	Neuroinflammation and neurovascular dysfunction targeting	Human patients (mild Alzheimer's disease)	Clinical: Randomized, controlled phase 2a, completed trial	Intravenous infusion of laromestrocel (25 M or 100 M cells; single or 4 monthly doses)	Rash et al. (2025)

*Note:* For each strategy, representative agents or methods, MSC sources tested (including bone marrow‐, adipose‐, umbilical cord‐, and endometrium‐derived populations), and the principal molecular targets or signaling pathways involved (e.g., OSKM factors, DNA methylation, TERT regulation, SIRT signaling, PI3K/AKT, and senescence‐associated pathways) are summarized.

The major scientific databases, including PubMed and Google Scholar, were consulted in the preparation of this manuscript. The literature search was conducted using relevant keywords, employed both individually and in combination through advanced search strategies, as well as using extended search strings equivalent to full titles, where appropriate. Priority was given to recent publications. Review articles indexed on PubMed were included to support a comprehensive and integrated view of the topic.

## 2 MSC Heterogeneity and Translational Implications

MSC heterogeneity represents a major challenge for clinical translation, as MSCs cannot be considered a uniform therapeutic product. Their phenotype and functional potency are influenced by donor‐related factors, including age, sex, body mass index and health status, as well as by tissue source, isolation procedures, culture conditions and passage number (Siegel et al. 2013; Česnik and Švajger 2024). This issue is particularly relevant for perinatal MSCs, which offer the advantage of being derived from developmentally young tissues, but still display considerable donor‐ and source‐dependent heterogeneity. Although the ISCT minimal criteria remain essential for MSC identification, based on plastic adherence, expression of CD105, CD73 and CD90, lack of hematopoietic markers and trilineage differentiation capacity, these parameters are not sufficient to predict therapeutic efficacy or responsiveness to rejuvenation strategies (Dominici et al. 2006). Therefore, MSC characterization should move beyond identity markers and incorporate mechanism‐of‐action‐related functional potency assays, which are critical to support batch comparability, release criteria and regulatory evaluation during clinical translation (Mendicino et al. 2014; Galipeau et al. 2016).

In this context, senescence is not only a biological limitation but also a manufacturing and product‐quality issue. During in vitro expansion, MSCs may progressively lose proliferative capacity, clonogenicity, differentiation potential, and immunomodulatory function. However, the onset and severity of senescence may vary substantially among donors and culture protocols, indicating that passage number alone should not be considered an adequate surrogate for cellular age. Donor health status is also highly relevant, as metabolic stress can accelerate MSC aging independently of chronological age. For instance, obesity has been shown to induce senescence in MSCs derived from bone marrow, subcutaneous adipose tissue, and visceral adipose tissue of young mice, supporting the concept that pathological metabolic conditions may impair MSC fitness even in young donors (Alessio et al. 2020).

These aspects are especially important for GMP‐compliant manufacturing. Clinical‐grade MSC production requires standardized donor screening, controlled isolation and expansion protocols, qualified reagents, validated cryopreservation procedures and predefined release criteria, including identity, purity, viability, sterility, genomic stability and potency. Large‐scale MSC manufacturing is further complicated by the intrinsic heterogeneity of MSC cultures and by process‐related variability, which may affect reproducibility and product comparability (Sanz‐Nogués and O'Brien 2021; Lechanteur et al. 2021).

## 3 Rejuvenation and Senescence

Senescence is the dynamic process in which healthy cells progressively lose their functionality, undergo permanent cell cycle arrest and acquire a distinctive secretory phenotype (Coppé et al. 2010; Siraj et al. 2023). In MSCs, senescence manifests with a “scrambled egg” morphology, a downregulation of markers such as CD105 and CD90 and an upregulation of CD264 and CD26. Aging cells shift towards a proinflammatory phenotype participating in inflammaging (Saliev and Singh 2025b). At the molecular level, senescent cells can be identified through several features, including altered morphology, upregulation of cell cycle inhibitors (p53, p21, p16), changes in the retinoblastoma protein pathway, increased senescence‐associated β‐galactosidase (SA‐β‐gal) activity, telomere shortening, and lipofuscin accumulation (Siraj et al. 2023).

First discovered by Hayflick and Moorhead in 1961, senescent cells resist apoptosis despite the hostile, pro‐apoptotic microenvironment created through the Senescence Associated Secretory Phenotype (SASP). This secretory profile includes pro‐inflammatory cytokines (IL‐6, IL‐8), chemokines, and proteases that can induce senescence and overall tissue damage in nearby cells through paracrine interactions. The survival of senescent cells is ensured by the upregulation of Senescent Cell‐Anti‐Apoptotic‐Pathways (SCAP) as shown in transcriptomic analysis (Kirkland and Tchkonia 2020). Relevant studies on MSCs show that it is possible to modulate senescence in this cell type using senotherapeutic agents. In particular, senolytic agents induce apoptosis in senescent cells, while senomorphic strategies aim to modulate the SASP and inflammation, restoring the tissue functionality (these approaches will be discussed in detail in Section 3.4).

Cellular rejuvenation aims to restore the biological state of cells, slowing their functional decline and reversing molecular hallmarks of aging.

In the understanding of cellular senescence and our ability to manipulate the aging process, advances such as the use of Yamanaka factors (Oct4, Sox2, Klf4 and c‐Myc—OSKM) and improvements in gene editing technologies represent an important milestone. In fact, cellular reprogramming strategies address fundamental mechanisms underlying aging process, such as genome instability and epigenetic modifications, telomere attrition, and alterations of the mitochondrial metabolism that physiologically occur in cells (Saliev and Singh 2025b).

### 3.1 Epigenetic Modification

Epigenetic modifications target cellular rejuvenation through different aging‐related layers: histone variants and their modifications, DNA methylation clock, nucleosome remodeling and changes in transcriptional signatures or in noncoding RNA profiles (Ji et al. 2023).

#### 3.1.1 Partial Reprogramming

Partial reprogramming enables chromatin remodeling and reversal of many hallmarks of aging, while preserving the original cell line and its functions. This strategy is based on the transient and cyclic expression of OSKM factors, which activates epigenetic rejuvenation programs without causing the complete dedifferentiation typical of total reprogramming (Cipriano et al. 2024). Through these controlled cycles, partial reprogramming can improve the functions of aged cells by enhancing DNA repair mechanisms and reestablishing expression profiles more similar to those of young cells.

Partial reprogramming requires extremely precise control of gene expression cycles, because unregulated activation can lead to dedifferentiation and potentially to tumors (Saliev and Singh 2024).

Delivery systems described in the literature include regulable adenoviral and inducible lentiviral platforms (Lehmann, Canatelli‐Mallat, Chiavellini, Cónsole, et al. 2019), which allow controlled expression of OSKM. In contrast, studies specifically targeting MSCs predominantly rely on Sendai virus vectors, which provide transient reprogramming without genomic integration (Ivanova et al. 2024).

Although viral vectors retain high efficiency, they pose significant risks for in vivo applications due to potential immunogenicity and off‐target effects. Inducible systems such as Tet‐On/Tet‐Off promoters improve temporal control but do not fully eliminate these risks (Lehmann, Canatelli‐Mallat, Chiavellini, Morel, et al. 2019; Saliev and Singh 2024). For this reason, nanoparticles and liposomes are being investigated despite their limited penetration of biological barriers (Saliev and Singh 2024).

#### 3.1.2 Small Molecules

Epigenetic modification can regulate chromatin accessibility and subsequent gene expression, influencing differentiation capacity and re‐activation of stemness genes (Ji et al. 2023). These mechanisms include DNA methylation catalyzed by DNA methyltransferases, histone modifications, such as acetylation, methylation, ubiquitination, and other post‐translational modifications, ATP‐dependent chromatin remodeling, and regulation mediated by ncRNAs (Walewska et al. 2023).

‘Epigenetic drugs’ can modulate enzymes of the epigenetic machinery through cells rejuvenation, increasing their clinical application (Völker‐Albert et al. 2020). In this category we can mention DNA‐methyltransferase inhibitors such as 5‐azacitidine (AzaC) and 5‐aza‐2′‐deoxycytidine (AzadC), bromodomain and extra‐terminal inhibitors that repress transcriptional elongation of acetylated chromatin, histone methylation inhibitors, and histone deacetylase (HDAC) inhibitors such as valproic acid (VPA) (Ji et al. 2023).

In vitro, epigenetic agents such as AzadC and HDAC inhibitors can modulate the immunoregulatory properties of MSCs and influence their differentiation potential (Walewska et al. 2023; Völker‐Albert et al. 2020). AzadC significantly increases the expression of HLA‐G1 and HLA‐G3 in bone marrow MSCs (BM‐MSCs) and adipose tissue MSCs (ASC‐MSCs), suggesting a role for demethylation in regulating HLA‐G expression increasing tolerability when transplanted (Teklemariam et al. 2014). AzaC alone has not proved to be sufficient to induce stable reprogramming: in Wharton's jelly MSCs (WJ‐MSCs), the expression of key pluripotency genes is, in fact, only transiently increased, with spontaneous regression within 72 h (Ong et al. 2021). In contrast, in ASC‐MSCs from elderly donors, treatment with AzaC has shown great results: an increase in the proliferation rate, greater resistance to reactive oxygen species (ROS), a reduction in senescent cells and superior resistance to apoptosis, effects consistent with a potential application in cellular rejuvenation strategies (Schellnegger et al. 2024; Kornicka et al. 2017).

The differing effects of AzaC on MSCs derived from different tissue sources could be explained by distinct epigenetic profiles, as well as by intrinsic donor‐dependent variability, which may influence responsiveness to this demethylating agent.

VPA instead shows a dose and context dependent effect on MSCs derived from different regions of the umbilical cord. In umbilical cord MSCs (UC‐MSCs) VPA has been used to promote the differentiation into hepatic cells, through the activation of the ERK and AKT pathways (An et al. 2014), and preconditioning with 2 mM VPA has been shown to enhance protein synthesis and cellular remodeling shifting the secretome towards a more neurotrophic profile (Işıldar et al. 2024). In cord blood MSCs (CB‐MSCs), short exposures to low doses of VPA (< 5 mM, 3 h) increase migratory capacity and enhance differentiation towards myogenic, chondrogenic and osteogenic lineages (Marquez‐Curtis et al. 2014). In WJ‐MSCs instead, VPA differentially regulates pluripotency or neural commitment depending on both dose and culture medium (Salami et al. 2019).

Overall, VPA emerges as a versatile epigenetic modulator capable of enhancing stemness, migration, or lineage specification, with effects strongly influenced by tissue of origin and culture conditions. This apparent dichotomy could be explained by the mechanism of action of VPA as an HDAC inhibitor, which causes a non‐specific increase in chromatin accessibility, leading to the activation of various transcriptional programs depending on the cellular context, dose, and duration of exposure.

#### 3.1.3 Gene Editing Approaches

CRISPR can be used both to understand the role of genes (p16INK4a or p53 pathways) in senescence and to silence them, delaying or reversing aging effects in targeted tissues thanks to the use of tissue‐specific promoters (Saliev and Singh 2024; Jia et al. 2024; Wagner and Wagner 2022; Konstantinou et al. 2024).

CRISPRa and CRISPR‐KO screening in MSCs identified SOX5 as a rejuvenating factor capable of activating HGMB2 and reducing senescence, and H2AZ1 as a pro‐senescence histone variant, whose depletion restores proliferation and youthful markers through depression of pleiotrophin (Li et al. 2025; Jing et al. 2023). These studies demonstrate how CRISPR enables the systematic mapping of the networks that regulate the senescent fate of MSCs.

CRISPR technology can be used to upregulate genes that enhance cellular resilience, repair mitochondrial DNA mutations, mediate the repair of double strand breaks or base excision repair (Saliev and Singh 2024; Chen, Hu, et al. 2024; Chen, Du, et al. 2024).

In this context CRISPR‐Cas9 can increase the efficacy of MSCs in regenerative medicine, particularly in inflammatory diseases or age‐related diseases, by activating telomeres and boosting the resilience towards oxidative stress and the inflammatory environment (Saliev and Singh 2024; Liao et al. 2024). Recent studies show that the engineering of MSCc can generate senescence‐resistant mesenchymal progenitors. Gorbunova and Seluanov (2025) demonstrates that genetic modification of FOXO3, through mutation of two critical phosphorylation sites, produces senescence‐resistant cells (SRCs) capable of withstanding senescence, oxidative stress and malignant transformation (Gorbunova and Seluanov 2025). The use of these cells in preclinical trials on chimpanzees via systemic infusion reported SRCs as safe, non‐immunogenic, and non‐tumorigenic, capable of reducing tissue senescence and biological age with regression of inflammation, oxidative stress, and DNA damage (Lei et al. 2025). These effects appear to be mediated by exosomes produced by SRCs, which are capable of restoring cellular homeostasis.

However, it is important to acknowledge that CRISPR techniques still face possible off‐target modifications, delivery difficulties, the need for specialized facilities and expertise, high costs and significant genetic risks linked to the germline transmission (Saliev and Singh 2024).

### 3.2 Telomerese

Telomeres are protective structures at chromosome ends composed of TTAGGG repeats and associated proteins, which shortening during cell division serves as a biomarker of aging (Arellano et al. 2024). Telomere length is regulated by telomerase, active in progenitor and stem cells but downregulated after differentiation, contributing to stem cell pool exhaustion and tissue aging (Jiang et al. 2025).

Critically short telomeres induce genomic instability, leading to senescence or apoptosis. Oxidative stress and inflammation accelerate telomere attrition (Zhang et al. 2016): mitochondrial dysfunction increases ROS production, which preferentially damages G‐rich telomeric sequences and amplifies shortening (Serakinci et al. 2004).

MSCs provide an important model for studying telomere dynamics and their impact on cellular aging. Fetal MSCs retain telomerase activity to varying degrees depending on tissue origin (Jiang et al. 2025; Trivanović et al. 2015) they still undergo senescence in culture, resulting in reduced functionality and increased inflammation (Jiang et al. 2025; Serakinci et al. 2004). To overcome the limited proliferative capacity of MSCs, several telomerase‐based strategies have been explored. Exogenous TERT alone enhances survival but is insufficient to fully immortalize MSCs (Jiang et al. 2025; Balducci et al. 2014). Co‐transduction of TERT with viral oncogenes such as HPV E6/E7 can achieve long‐term expansion but carries risks of genomic instability (Jiang et al. 2025; Tsai et al. 2010).

In vitro non‐viral approaches, combining TERT overexpression with p53 silencing, promote stable proliferation (Jiang et al. 2025; Rakhmatullina et al. 2023). Additionally, dual expression of TERT and VEGF not only prolongs replicative lifespan but enhances the angiogenic potential of MSCs (Jiang et al. 2025; Tang et al. 2013).

Moreover, the use of antioxidant compounds has been tested in vitro on MSCs to boost proliferation and telomerase activity. Pistachio green pericap extract increases cell viability of BM‐MSCs reducing the rate of senescence (Askari et al. 2023). In the same way, L‐carnitine on ASC‐MSCs changes the methylation status in the hTERT promoter decreasing cell aging (Askari et al. 2023; Farahzadi et al. 2018) and curcumin in mouse ASC‐MSCs increased the expression of TERT (Askari et al. 2023; Pirmoradi et al. 2018).

The use of antioxidant and anti‐inflammatory agents for telomere attrition has been studied also in vivo with great results, showing how vitamin C (Cai et al. 2023; Qun et al. 2009), vitamin E (Corina et al. 2018), resveratrol (Opstad et al. 2021; Amano et al. 2019) and spermidine (Gabandé‐Rodríguez et al. 2020) can mitigate oxidative damage, reduce chronic inflammation, and ultimately slow down telomere shortening (Schellnegger et al. 2024) Another in vivo study further supports the potential of telomerase‐based rejuvenation: the use adeno associated virus mediated TERT gene therapy to counteract systemic aging has been approved (NCT04133649) (Ji et al. 2023; Davidsohn et al. 2019).

### 3.3 Metabolic Rejuvenation

ROS are a hallmark of advanced aging, together with the accumulation of mtDNA mutations, which arise as a consequence of ROS‐induced damage and the lack of efficient DNA repair mechanisms within mitochondria (Chistiakov et al. 2014; Phua et al. 2023; Schuliga et al. 2018; Branco et al. 2023). Defects in the oxidative phosphorylation (OXPHOS) system compromise the production of growth factors, thereby impairing stem cell self‐renewal and fate determination (Branco et al. 2023). Antioxidant‐based approaches may improve culture conditions, preserve cellular functionality, and delay the onset of replicative‐ and stress‐induced senescence. In this context, combined treatment with epigallocatechin gallate (EGCG) and sulforaphane has been shown to counteract in vitro oxidative stress in human amniotic fluid stem cells, reducing ROS accumulation, increasing GSH levels, and enhancing endogenous antioxidant defenses, while sustaining OCT4 and NANOG expression and modulating senescence‐associated markers. This evidence is particularly relevant in the perinatal stem cell field, as it supports the use of natural redox‐modulating compounds to delay culture‐induced stemness loss during ex vivo expansion (Marrazzo et al. 2018).

Treatment with calcitriol, the active form of vitamin D (1α,25(OH)_2_D₃) has shown to increase cell proliferation in BM‐MSCs. Calcitriol exerts anti‐aging effects by reducing oxidative stress and DNA damage by downregulating the p16/Rb and p53/p21 pathways (Klotz et al. 2012; Borojević et al. 2022). In addition, it negatively modulates p16/p19 signaling and attenuates the SASP (Borojević et al. 2022; Yang et al. 2020). Although additional mechanisms involving metabolic and inflammatory signaling have been recognized, many antioxidant and rejuvenating compounds converge on SIRT1‐related pathways. SIRT1 signaling pathway plays a role in aging and functionality of MSCs by contributing to protection against oxidative stress, DNA damage, apoptotic and inflammatory stimuli (Borojević et al. 2022; Zainabadi 2018).

Vitamin D modulates stemness and osteogenic commitment in BM‐MSCs mainly through SIRT1 signaling. Specifically, it promotes the expression of the pluripotency markers Nanog and Sox2, supporting self‐renewal and multilineage potential. In contrast, the effects of vitamin D on cell proliferation and senescence are largely SIRT1‐independent and mainly associated with the reduction of oxidative stress and DNA damage (Borojević et al. 2022).

Among the antioxidants, resveratrol, a polyphenolic phytoalexin, is known for its ability to delay cellular aging (Ji et al. 2023; Davidsohn et al. 2019). Resveratrol has shown great results in highly inflammatory environments by restoring osteogenic and extracellular matrix markers in inflamed periodontal ligament‐derived MSCs (Wang et al. 2018).

In this context, the hormone asprosin has beens hown to regulate the cell metabolism contributing to the aging process. Supplementation with its recombinant form gives MSCs functional rejuvenation, leading to enhanced proliferation and migratory capacity, reduced senescence‐associated cell cycle arrest, and improved DNA repair. These effects are mediated by glycolysis‐dependent epigenetic remodeling through H3K18 lactylation (Zhang et al. 2019; Zhang et al. 2026).

An alternative rejuvenation strategy involves enhancing DNA repair capacity through nicotinamide mononucleotide (NMN), a direct precursor of NAD^+^ and a cofactor of DNA repair enzymes, including sirtuins (Ji et al. 2023). In vitro expansion of MSCs is characterized by decreased NAD^+^ levels, reduced SIRT1 and SIRT3 function with a consequent increase of oxidative stress and senescence (Denu et al. 2016; Yuan et al. 2020). Replenishment of intracellular NAD^+^ levels restores mitochondrial fitness and autophagy/mitophagy in MSCs (Ji et al. 2023; Ruszkiewicz et al. 2022).

Moreover, combined treatment with resveratrol (activator of SIRT1) and AzaC upregulates mitochondrial quality control proteins such as PINK1 and Parkin, enhancing mitophagy and contributing to MSC rejuvenation (Kornicka et al. 2019).

Notably high levels of ROS act as intracellular messengers that regulate differentiation pathways. For this reason, differentiation protocols must be optimized to keep oxidative stress within a physiological range. In the perinatal context, vitamin C has been reported to enhance cardiogenic differentiation in amniotic fluid stem cells (AF‐SCs), while sesamin promotes the chondrogenic differentiation of AF‐SCs (Markmee et al. 2019).

### 3.4 Modulation of Senescence

The modulation of cellular senescence represents a key strategy to restore tissue homeostasis, counteract organismal aging, and reduce chronic inflammation. In this context, controlling senescence in MSCs is particularly relevant, as these cells play a crucial role in tissue maintenance and regeneration in bone, cartilage, and adipose tissue (Kirkland and Tchkonia 2017; Kirkland et al. 2017; Galderisi et al. 2022).

As introduced in Section 3, senescent cells are characterized by the acquisition of a SASP, which promotes inflammation and can propagate senescence to neighboring cells through paracrine mechanisms and reinforce the senescence process through autocrine mechanisms, and increase in SCAPs (Coppé et al. 2010; van Deursen 2014). These features make senescent cells important therapeutic targets. Two main classes of senotherapeutic approaches have been developed to target senescent cells.

#### 3.4.1 Senolytic Therapies

Senolytic agents selectively induce apoptosis in senescent cells by targeting pro‐survival mechanisms, including SCAPs. Key regulators involved in these pathways include members of the BCL‐2/BCL‐XL family, PI3K/AKT signaling, and ephrin‐mediated survival networks (Kirkland and Tchkonia 2017; Mazzone et al. 2025). In silico studies have identified several key pathways that can be targeted for senolytic drugs. Further analysis using siRNA was conducted to validate the effect of targeting SCAPs, thereby with the aim of reducing the SASP. Pivotal regulators involved in senescence are members of the BCL‐2/BCL‐XL family, the PI3Kδ/AKT signaling pathways, and components of ephrin‐mediated survival pathways involving multiple tyrosine kinases. Additional factors include p21 and plasminogen activator inhibitors 1 and 2 (PAI‐1 and PAI‐2) (Goldschneider and Mehlen 2010; Zhu et al. 2015).

Dasatinib is a tyrosine kinase inhibitor approved for oncological use that has proven to be effective in clearing human senescent adipocyte progenitors (Zhu et al. 2015). It promotes apoptosis mediated by dependence receptors, including ephrins, addressing a key signaling pathway of senescence (Kirkland and Tchkonia 2020).

Dasatinib in combination with Quercetin (a natural flavonoid; D + Q) is found to be particularly powerful since it addresses several key nodes of the SCAP network, aiding the elimination of a wider range of senescent cells in comparison to the use of the single agents alone (Kirkland and Tchkonia 2020). D + Q reduces SA‐β‐gal activity and significantly improves their proliferative capacity, as well as restoring cytoskeletal integrity. A recent application involving UC‐MSCs from preeclampsia (PE) patients treated with the combination of these two agents showed promising results (He et al. 2025). In vitro, D + Q‐treated old BM‐MSCs show reduced total cell number due to increased apoptosis, decreased SA‐β‐gal positivity, reduced expression of inflammatory and senescence‐associated markers, and enhanced proliferation rate and osteogenic differentiation potential (Zhou, Xin, et al. 2021).

In vivo, systemic administration of D + Q in aged immunodeficient mice restores the formation of newly mineralized bone to levels comparable to those observed in young mice. The regenerated bone displays a structure resembling physiological conditions, characterized by increased marrow space and a balanced osteoclast/osteoblast ratio. This suggests that the enhancement of osteogenesis may result from the clearance of senescent cells, which exhibit limited osteogenic potential that also adversely affect the surrounding microenvironment through SASP and associated inflammatory signaling (Zhou, Xin, et al. 2021).

Moreover, this combination shows strong clinical potential, as it is currently evaluated in Phase I/II trials for several age‐related conditions: idiopathic pulmonary fibrosis, diabetic kidney disease, Alzheimer's disease, osteoporosis, and frailty (Česnik and Švajger 2024).

Quercetin alone has also been studied to fight aging and functional decline in BM‐MSCs by counteracting oxidative stress while restoring epigenetic stability by inhibiting PI3K, various other kinases, and serpins (Sun et al. 2025).

An analog of Quercetin is Fisetin, a naturally occurring bioactive flavonoid found in various fruits and vegetables that has been identified as a senolytic drug. Fisetin acts in part by inhibiting members of the BCL‐2 family as well as HIF‐1α (Kirkland et al. 2017). Like Quercetin, Fisetin has proven to selectively reduce viability of senescent Human Umbilical Vein Endothelial Cells (HUVECs) (Zhu et al. 2017) and most recently has proved to attenuate senescence in ASC‐MSCs during culture expansion (Mullen et al. 2023). In combination with Losartan, an angiotensin receptor II blocker, has also revealed a significant anti‐inflammatory and anti‐oxidative activity in BM‐MSCs aiding their rejuvenation (Nishimura et al. 2025).

#### 3.4.2 Senomorphic Therapies

The use of therapies that induce apoptosis in senescent cells is not always appropriate, particularly in physiological contexts where senescent cells play beneficial roles in tissue remodeling, wound healing, and immunosurveillance. In such cases, senomorphic approaches are preferred, as they aim to preserve these functions while limiting detrimental effects (Saliev and Singh 2025).

Senomorphic strategies act by modulating the senescent phenotype without eliminating senescent cells. They attenuate the SASP‐induced inflammation and restore tissue functionality by enhancing the regenerative capacity of aged tissues. The SASP consists of a secretome composed of pro‐inflammatory cytokines, chemokines, growth factors, and anti‐apoptotic factors, which contribute to tissue dysfunction and the propagation of senescence (Coppé et al. 2010; Siraj et al. 2023). The composition of the SASP evolves over time. Early senescence is associated with pro‐fibrotic and immunomodulatory factors, including TGF‐β, VEGF, and extracellular matrix components, which support tissue remodeling (Huang et al. 2022). In contrast, chronic senescence is characterized by the predominance of pro‐inflammatory cytokines and proteases, contributing to tissue dysfunction. This temporal heterogeneity suggests that senomorphic interventions should consider the timing of senescence onset to maximize therapeutic efficacy. Consequently, therapeutic strategies should aim at selective modulation rather than complete suppression of SASP. For example, targeting key inflammatory mediators such as IL‐1β, IL‐6, and matrix metalloproteinases allows preservation of regenerative signals while limiting pathological inflammation (Alqahtani et al. 2025).

At the molecular level, SASP production is tightly regulated by stress‐responsive signaling pathways and transcription factors, including NF‐κB, C/EBPβ, mTOR, JAK/STAT, and p38 MAPK (Ya and Bayraktutan 2024).

Among senomorphic agents, rapamycin (RAPA), an immunosuppressive antibiotic, modulates cellular senescence by inhibiting the Akt/mTOR pathway, reducing ROS production and regulating pluripotency‐associated genes such as Oct‐4 and Nanog. In senescent UC‐MSCs, RAPA treatment restores angiogenic potential both in vitro and in vivo. Specifically, it reduces senescence markers (SA‐β‐gal positivity and p53 expression) while partially recovering MSC phenotype and function. When transplanted into a murine model of limb ischemia, RAPA‐treated senescent MSCs significantly improved neovascularization and resulted in limb salvage in approximately 50% of cases, with vascular density (CD31^+^ vessels) comparable to healthy controls (Cao et al. 2022). These findings, together with the evidence that RAPA in placenta‐derived MSCs can modulate IL‐6 and IL‐8 and reduce senescence markers through mTOR inhibition suggest that RAPA can functionally rejuvenate senescent MSCs and enhance their therapeutic efficacy (Sheppard et al. 2024).

A central regulator of SASP expression is the NF‐κB pathway, which drives the transcription of pro‐inflammatory cytokines reinforcing the senescent state. IL‐1α acts as an upstream activator of this axis in senescent MSCs, promoting NF‐κB activation and subsequent secretion of IL‐6 and IL‐8. Inhibition of IL‐1α or NF‐κB represents a prototypical senomorphic strategy (Meng et al. 2023; Saliev and Singh 2025). In muscle‐derived stem/progenitor cells (MSC‐like populations), the use of NF‐κB inhibitors has a rejuvenating effect on aged mouse cells, restoring differentiation potential and stress resistance (Proto et al. 2017).

Another important class of senomorphic agents includes JAK inhibitors (ruxolitinib and baricitinib), which suppress the inflammatory signaling of SASP cytokines (Politano et al. 2025; Hu et al. 2025). In preclinical models and early clinical studies, JAK inhibition has been shown to reduce systemic inflammation, improve hematopoietic function, and restore tissue homeostasis in aged organs (Kim et al. 2024; Saliev and Singh 2025).

Finally, natural compounds such as polyphenols exhibit dose‐dependent senotherapeutic effects. At low concentrations, they act as senomorphics by reducing oxidative stress, preventing the onset of senescence, and modulating SASP‐related signaling, in some cases contributing to partial reversal of the senescent phenotype. Conversely, at higher doses, they can increase intracellular ROS levels and induce apoptosis, thereby exerting senolytic activity (Mazzone et al. 2025).

**FIGURE 2:**
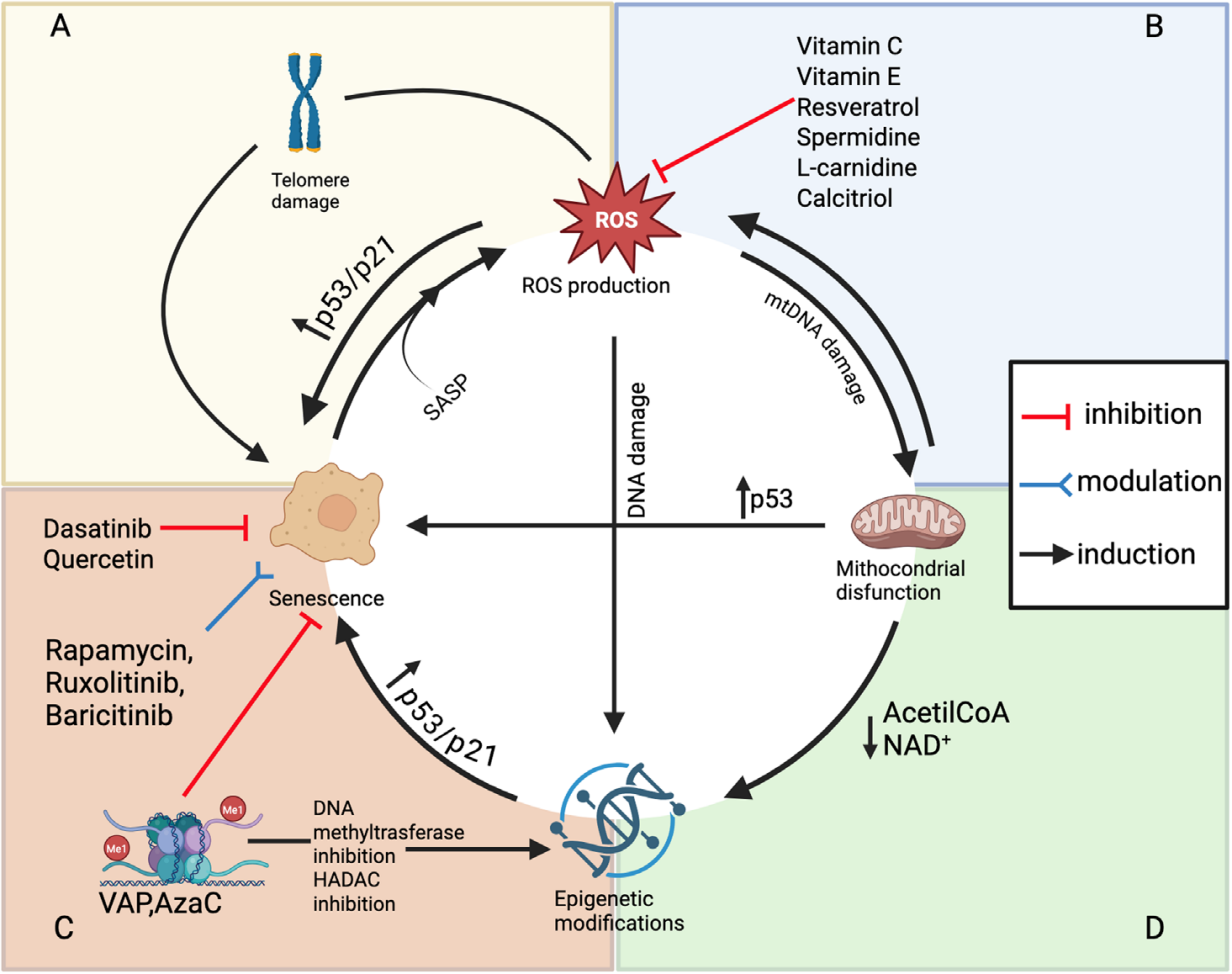
Interplay between mitochondrial dysfunction, genomic instability, epigenetic remodeling, and cellular senescence. (A) ROS‐senescence axis: Increased ROS production induces oxidative damage to telomeres, promoting cellular senescence. Furthermore, ROS overproduction activates the p53/p21 signaling pathway, enhancing the senescence process. Conversely, senescent cells secrete SASP factors which stimulate ROS production, establishing a positive feedback loop. (B) ROS‐Mitochondrial damage axis: Mitochondrial dysfunction promotes ROS overproduction and redox imbalance, enhancing DNA damage and stress responses. Antioxidants and metabolic modulators (e.g., vitamin C, vitamin E, resveratrol, spermidine, L‐carnitine, and calcitriol) may counteract ROS accumulation and restore cellular homeostasis. (C) Senescence‐Epigenetic modification axis: Epigenetic alterations can be induced by compounds such as azacytidine (AzaC) and valproic acid (VPA), which inhibit DNA methyltransferases and histone deacetylases, respectively. The epigenetic changes may promote cellular senescence through activation of the p53/p21 signaling pathway. Cellular senescence can be modulated by senomorphic agents (e.g., rapamycin, ruxolitinib, and baricitinib), which attenuate senescence‐associated phenotypes, or selectively eliminated by senolytic agents (e.g., dasatinib and quercetin). (D) Mitochondrial damage‐Epigenetic modification axis: Mitochondrial sfunction can cause epigenetic modifications by altering cellular metabolism and redox balance. In particular, it decrease availability of key metabolites such as NAD^+^ and acetyl‐CoA, which act as cofactors for chromatin‐modifying enzymes. These lead to abnormal DNA methylation and histone modification patterns.

Taken together, these pathways form a highly interconnected network rather than acting as isolated processes, as illustrated in Figure 2.

### 3.5 Extracellular Vesicles

MSCs exert their beneficial effects also through their secretome, which consists of growth factors, cytokines, chemokines, hormones, and extracellular vesicles (EVs) (Zhang et al. 2025). EVs are heterogeneous, lipid‐bilayer‐delimited particles released by cells and spanning the nanometer‐to‐micrometer size range. They may originate from endosomal compartments or bud directly from the plasma membrane and carry bioactive molecules, including proteins, lipids, and nucleic acids (Khanh et al. 2020; Keshtkar et al. 2018; Seo et al. 2019). EVs secreted by MSCs recapitulate many of the biological functions of the derived cells like the release of anti‐inflammatory, migratory, and angiogenic factors, leading to their investigation for age‐related inflammatory and degenerative diseases (Lan et al. 2025). Compared to viable cells, EVs offer numerous advantages: they are considered safer, as they are unable to replicate; they exhibit low immunogenicity and high biocompatibility. Moreover, EVs can be stored for long periods with relatively low production costs and can be administered via various routes (Kou et al. 2022; Draguet et al. 2023; Zhang et al. 2025). EVs interact with target cells through fusion with the plasma membrane, endocytic internalization followed by cargo release, and receptor‐ligand interactions that activate intracellular signaling pathways (Miclau et al. 2023).

Notably, in vitro, EVs derived from young MSCs have been shown to counteract age‐associated dysfunctions in adult MSCs by restoring redox balance, and ultimately limiting cellular senescence through the transfer of antioxidant miRNAs, mRNAs and proteins (Khanh et al. 2020). In addition, UC‐MSC‐EVs can promote cell cycle re‐entry by transferring proliferating cell nuclear antigen (PCNA) to adult BM‐MSCs, resulting in enhanced proliferation, telomere length maintenance, wound healing capacity and angiogenesis (Lei et al. 2021). Beyond rejuvenation, MSC‐derived EVs have also emerged as potential senotherapeutic agents, as they are able to attenuate the SASP, partly through the normalization of the IL‐6RA/STAT3 signaling axis as demonstrated with the use of Amniotic membrane‐EVs on MIN16 (Xiao et al. 2026).

Early‐phase clinical studies have reported potential benefits of EV‐BM‐MSCs in conditions such as Alzheimer's disease, with modest improvements in cognitive performance and brain volume (Rash et al. 2025). There are currently about 50 clinical trials registered on ClinicalTrials.gov using the search term “mesenchymal stem cell‐derived extracellular vesicles.” Emerging evidence from preclinical studies and early‐phase clinical trials suggests that MSC‐derived extracellular vesicles exert therapeutic effects across multiple pathological conditions, including COVID‐19, respiratory inflammatory diseases, myocardial injury, spinal cord injury, and skin regeneration (Zhang et al. 2025). However, some clinical studies have not shown significant benefits compared to placebo, highlighting the need for further studies to confirm their efficacy and better define their therapeutic applications.

The clinical translation of MSC‐based EVs therapies still faces several challenges, including genetic instability, reduced efficacy in hostile microenvironments, difficulties in large‐scale production due to changes in cell morphology and a decrease in proliferation rates, and a short half‐life, along with culture conditions that hinder their therapeutic potential (Zhou, Yuan, et al. 2021; Mai et al. 2021; Costa et al. 2021). Furthermore, standardization is hindered by the heterogeneity of the vesicles due to variations in donor characteristics (Zhou, Yuan, et al. 2021) as well as the tissue from which they were isolated. Among the various challenges to translational EVs application the lack of a standardized method for separation and purification needs to be mentioned. The 2D cell supernatant collection system containing EVs increases the risk of contamination, and ultracentrifugation, in addition to being an open system, introduces extra steps, lacks scalability, and is time‐consuming (Brennan et al. 2020). For this reason, automated 3D closed systems are currently preferred for collecting EVs, as they are more scalable, cost‐effective, and time‐efficient, while offering increased cell viability (Russell et al. 2018; Hanley et al. 2014) and scalable models that meet cGMP requirements for sterility (Wiest and Zubair 2025).

## 4 Conclusions

To enhance the translational potential of cell‐based therapies for in vivo applications, it is crucial to have high‐quality, expansible cells as starting material. Counteracting culture‐induced replicative senescence distinct from donor‐ and disease‐related aging may preserve MSC proliferation, clonogenicity, differentiation, immunomodulatory activity, and secretory profile. This is also a manufacturing priority because donor characteristics, tissue source, and culture conditions can affect potency, batch comparability, and product consistency. Rejuvenation should therefore be intended not as a complete return to a young state, but as the recovery of therapeutically relevant functions without loss of lineage identity.

Moreover, studying rejuvenation therapies could lead to interesting insights into the molecular mechanisms underlying aging, shedding light on key regulatory pathways of homeostasis.

However, the available approaches differ substantially in their mechanisms of action, degree of experimental control, and safety profile. Despite encouraging preclinical findings, concerns related to genomic stability, tumorigenicity, delivery, and inter‐donor and inter‐protocol variability continue to limit the clinical translation of selected strategies, particularly those involving genetic manipulation.

Rejuvenation technologies may be relevant across a broad range of pathological settings, including tissue regeneration, diabetes, bone degeneration, neurodegenerative and cardiovascular diseases, chronic lung and kidney disorders, fibrosis, autoimmune and inflammatory diseases, and cancer. However, the gap between promising preclinical findings and consistent clinical outcomes remains substantial. Future research should therefore focus on several key areas: first, the adoption of harmonized criteria to define MSC rejuvenation beyond increased proliferation, integrating cellular identity, senescence‐associated markers, genomic stability, mitochondrial function, secretome composition, and disease‐relevant functional activity; second, the systematic comparison of rejuvenation approaches across different MSC sources, donor profiles, and culture conditions; third, the development of mechanism‐of‐action‐based potency assays that can support batch comparability and product release; and finally, the evaluation of long‐term safety, including genomic stability, tumorigenicity, persistence, and functional behavior after transplantation.

Rather than identifying a universally optimal rejuvenation strategy, future studies should determine which interventions are most appropriate for specific MSC sources, manufacturing platforms, and therapeutic indications. In this perspective, the integration of standardized GMP‐compatible workflows with robust in vitro and in vivo validation will be essential to establish whether the functional improvements induced by rejuvenation are durable, reproducible, and clinically meaningful. This indication‐specific and product‐oriented approach may help bridge the gap between mechanistic advances in MSC aging research and the development of reliable MSC‐based therapeutic products.

## Author Contributions

Conceptualization, F.P. and F.A; methodology, A.R; investigation, A.S. and I.B; writing original draft preparation, A.S. and I.B; writing review and editing, F.P, A.R, M.R., L.M., F.A., S.R. and P.L.; visualization, P.L.; data curation, A.S.; supervision, F.A. and S.R.; project administration, F.A.; funding acquisition, F.A. All authors have read and agreed to the published version of the manuscript.

## Funding

This work was supported by the Associazione Giovani Diabetici AGD BOLOGNA.

## Ethics Statement

The authors have nothing to report.

## Conflicts of Interest

The authors declare no conflicts of interest.

## Data Availability

The authors have nothing to report.
